# Compartmentalization of Resistance-Associated Substitutions in HIV/HCV-Infected Patients: Possible Correlation with Infecting HCV Genotype

**DOI:** 10.3390/v13081486

**Published:** 2021-07-29

**Authors:** Giulia Morsica, Riccardo Vercesi, Hamid Hasson, Emanuela Messina, Caterina Uberti-Foppa, Sabrina Bagaglio

**Affiliations:** 1 Division of Infectious Diseases, Scientific Institute Ospedale San Raffaele, 20132 Milan, Italy; r.vercesi93@gmail.com (R.V.); hasson.hamid@hsr.it (H.H.); messina.emanuela@hsr.it (E.M.); uberti.caterina@hsr.it (C.U.-F.); sabrinabagaglio@hotmail.com (S.B.); 2 Faculty of Medicine and Surgery, Vita-Salute University, 20132 Milan, Italy

**Keywords:** liver, plasma, compartmentalization, resistance, HCV, HIV, treatment, direct-acting antivirals, genotype

## Abstract

Resistance-associated substitutions (RASs) may exist prior to treatment and contribute to the failure of treatment with direct-acting antivirals (DAAs). As the major site of HCV replication, naturally occurring variants with RASs may segregate into the liver. In the present study, we performed viral population sequencing to retrospectively investigate the NS3 and NS5A RAS profiles in 34 HIV/HCV coinfected patients naïve to anti-HCV treatment who underwent diagnostic liver biopsy between 2000 and 2006 and had liver and plasma samples available. Sixteen were infected by HCV genotype (GT) 1a, 11 by GT3a, and 7 by GT4d. The analysis of the NS3 domain in GT1a showed a difference in strain between the liver and plasma in three cases, with a preponderance of specific RASs in the liver compartment. In GT4d samples, 6/7 coupled liver and plasma samples were concordant with no RASs. Sequence analysis of the NS5A domain showed the presence of RASs in the livers of 2/16 patients harboring GT1a but not in the corresponding plasma. In GT4d, NS5A RASs were detected in 7/7 liver tissues and 5/7 plasma samples. NS3 domain and NS5A domain were found to be conserved in plasma and livers of patients infected with GT3a. Thus, RASs within GT1a and GT4d more likely segregate into the liver and may explain the emergence of resistant strains during DAA treatment.

## 1. Introduction

Hepatitis C virus (HCV) is a major human liver pathogen infecting approximately 71 million people worldwide [1]. Approximately 2.3 million of these patients are coinfected with human immunodeficiency virus (HIV) [2]. With the extended life expectancy offered by combined antiretroviral therapy, this patient subpopulation is at increased risk of long-term complications from HCV-associated chronic liver disease, including cirrhosis and hepatocellular carcinoma. The development of direct-acting antiviral agents (DAAs) has dramatically improved the treatment options for HCV, and a sustained virological response (SVR) in HIV/HCV coinfected patients is achieved at rates similar to those of HCV monoinfected patients [3,4,5]. However, outside of clinical trials, the population of HIV/HCV coinfected patients may have risk factors that, by themselves or in combination, may lead to lower SVR [6].

Because of the lack of proofreading activity of the RNA-dependent RNA polymerase and the rapid turnover rate in HCV replication, HCV has an intrinsic predisposition to mutate, resulting in 67 confirmed subtypes of seven HCV genotypes [7] characterized by different geographic distribution, pathogenesis, and response to anti-HCV treatment.

The intrinsic genetic variability of HCV may be responsible for the emergence of a viral genome with resistance-associated substitutions (RASs) that confer decreased susceptibility to DAAs and/or cross-resistance with other inhibitors of the same class, rendering the treatment or retreatment with new antivirals less effective [8,9].

RASs found at failure generally develop during treatment with DAAs, but in some patients, they may pre-exist as naturally occurring variants before treatment. The emergence of a viral genome harboring RASs during DAA treatment is probably the consequence of the intrahepatic replicative advantage of pre-existing minor variants that became dominant under drug pressure.

The distribution of RASs according to targeted region is genotype-specific. As the genotype-specific RAS pattern may impair the efficacy of DAAs, the prevalence of naturally occurring RASs in relation to infecting genotype has been the focus of intense investigation [10,11,12,13]. However, little is known about the compartmentalization of HCV variants with natural resistance to anti-NS3 and anti-NS5A compounds in HIV/HCV coinfected patients according to HCV genotype. Previous reports [14,15,16] have invariably shown the presence of variants harboring RASs in the liver rather than the plasma.

In the present study, we investigated the possible compartmentalization of naturally occurring NS3 and NS5A RASs in the liver and plasma of HIV/HCV coinfected patients according to different HCV genotypes because diversification within these compartments could explain why certain patients have difficulty being cured.

## 2. Methods

### 2.1. Study Group

Of 100 HIV/HCV coinfected patients who underwent liver biopsy for diagnostic purposes during the years 2000–2006, 34 were selected on the basis of available paired plasma samples and paraffin-embedded liver tissue for molecular investigation and information on the HCV infecting genotype as assessed by routine laboratory test. Plasma samples had been collected from these 34 patients for immune and virological studies concerning HIV/HCV coinfection; therefore, paraffin-embedded liver tissues were chosen according to plasma sample availability. The histological pattern was assessed according to the Metavir score [17]. Epidemiological, clinical, and laboratory parameters were recorded in an internal database as part of routine clinical care.

The study was approved by the ethics committee of San Raffaele Hospital, Milan, Italy.

### 2.2. Sequence Analysis of the NS3 Protease and NS5A Domain

The liver and plasma samples were investigated using in-house protocols for HCV-RNA extraction, reverse transcriptase (RT) amplification, and population sequencing of the NS3 protease domain, comprising 181 amino acids (aa), and the NS5A domain, comprising 100 aa, as described previously [15,18].

### 2.3. RNA Extraction and Amplification

Total RNA was extracted from paraffin-embedded liver tissue samples using standard procedures as follows: Two 10 mm thick sections (1.5–2 cm in length) were homogenized in 1 mL of xylene and kept at room temperature for 5 min before centrifuging at 14,000 rpm for 5 min. The supernatant was discarded and 1 mL of ethanol was added before centrifuging again at 14,000 rpm for 5 min. The process was repeated using a descending series of ethanol concentrations until all of the paraffin was completely removed. The tissue samples were then washed twice with distilled RNAase-free water to remove any residual ethanol, treated with proteinase K (800 µg/mL), and incubated overnight at 56 °C. Finally, the QIAamp RNA Mini-kit (Qiagen SpA, Milan, Italy) was used following the manufacturer’s instructions. To investigate the RNA quality, glyceraldehyde-3-phosphate dehydrogenase (GAPDH) was amplified using the following primer sets: GAPDH-1 (sense primer) 5′-CCATGGAGAAGGCTGGGG-3′ (nucleotides 371–388), GADPH-2 (antisense primer) 5′-CAAAGTTGTCATGGATGACC-3′ (nucleotides 566–546).

Total RNA was extracted from plasma samples using the Qiagen Viral RNA Mini kit as recommended by the manufacturer (Qiagen, Hilden, Germany).

After total RNA extraction from liver and plasma and amplification as described previously [15,18] the PCR products obtained from the NS3 protease gene and NS5A domain were purified using a microcolumn system and directly sequenced on an ABI 3730 XL (Applied Biosystems, Life Technologies, Italy). Both sense and antisense strains were sequenced for each PCR fragment. Only results confirmed in at least two different experiments were considered in subsequent analyses.

### 2.4. Phylogenetic Analysis

The multiple alignments of the nucleotide sequences were inferred using Clustal_X version 164.b [19]. The nucleotide distances between sequences derived from different compartments were determined by generating a distance matrix using the DNADIST program (Phylip 3.5c package, Department of Genetics, University of Washington: Seattle, WA, USA) [20]. A phylogenetic tree was then constructed using NEIGHBOR (Phylip) with random additions and drawn using TreeViewPPC version 1.5.3 [21]. Bootstrap analysis with SEQBOOT (100 resamplings) was used to place approximate confidence limits on the individual node.

**Figure 1:**
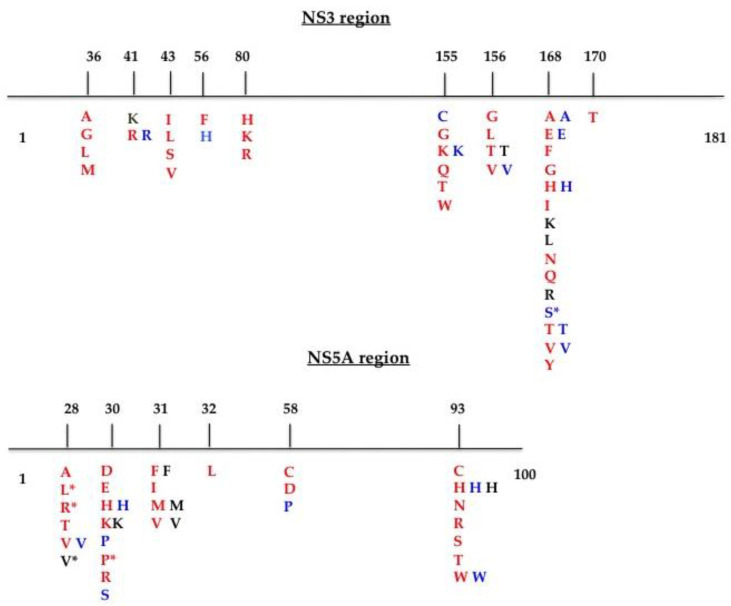
Resistance-associated substitutions within the NS3 (181 aa) and NS5A (100 aa) domains identified in the literature [13,14,15]. Amino acid (aa) substitutions are color-coded based on HCV genotype: GT1a, red; GT3a, black; GT4d, blue. * aa substitution at a scored position.

HCV genotypes obtained by routine laboratory testing were redetermined by alignment of the nucleotide sequences and phylogenetic analysis of the NS3 and NS5A domains with respective prototypes (for genotype 1a (GT1a), isolate NC004102; GT3a, isolate D17763; and GT4d, isolate DQ418786) and were confirmed by applying the Geno2Pheno (https://hcv.geno2pheno.org/2019, accessed on 1 April 2021) algorithm to the NS3 and NS5A sequences. The RAS profiles were determined from the literature [22,23,24] and by applying the Geno2Pheno HCV algorithm. A schematic representation of the RAS positions is shown in Figure 1. We also included and considered mutations at scored positions. The sequences were submitted to GenBank (NS3 domain accession numbers: MT863870–MT863937; NS5A region accession numbers: MT863938–MT864005).

### 2.5. Statistical Analysis

The descriptive data are presented as the number and percentage for categorical variables and as the median and interquartile range (IQR) for continuous variables. The characteristics of HIV-1-positive patients were compared using chi-square or Fisher’s exact test for categorical variables and the Wilcoxon rank-sum test for continuous variables.

For each RAS, absolute frequencies and percentages were calculated for both plasma and tissue samples. To evaluate the association between mutations and the infecting HCV genotype, we applied contingency tables (chi-squared test). Two-sided *p* values < 0.05 were considered significant.

Data were collected and tabulated using Microsoft Office Excel 2007. All statistical analyses were performed using GraphPad Prism Version 5.0a (GraphPad Software Inc., San Diego, CA, USA).

## 3. Results

### 3.1. Characteristics of HIV/HCV Coinfected Patients

The clinical characteristics of the HIV/HCV coinfected patients included in this study are summarized in Table 1 according to HCV infecting genotype (47% GT1a, 32.4% GT3a, and 20.6% GT4d). The majority of patients were male (70.6%). The main risk factor for HIV infection was intravenous drug use (IVDU). Seven (20.6%) patients had a histological diagnosis of advanced liver disease assessed by Metavir score (fibrosis degree ≥ F3), including two patients with cirrhosis.

The median alanine aminotransferase (ALT) and aspartate aminotransferase (AST) levels were in the upper normal range (Table 1). The patients had a relatively preserved immune status assessed by CD4 T cell count. Two patients were positive for the hepatitis B surface antigen (HBsAg), but 21/34 (61.8%) had serological markers of past HBV infection, including 11 who were anti-HBsAg positive and 10 with antibodies against HBV core antigen (anti-HBc). The remaining 11 (31.4%) patients had no serological markers of past or current HBV infection. HIV-RNA was ≥50 copies/mL in 23/34 (67.6%) patients.

No differences were found concerning the clinical characteristics of the study group according to HCV infecting genotype. In particular, the three genotype groups had a similar HCV load and immune status assessed by CD4 T cell count (Table 1).

### 3.2. Sequence Analysis of the NS3 and NS5A Domains

#### 3.2.1. Phylogenetic Analysis

The phylogenetic analysis of the NS3 domain showed that a number of sequences from paired liver and plasma samples were unrelated to one another. For example, the plasma sample from patient (PT) 15 was more strictly related to the plasma sample of PT24 than to paired liver tissue.

**Figure 2:**
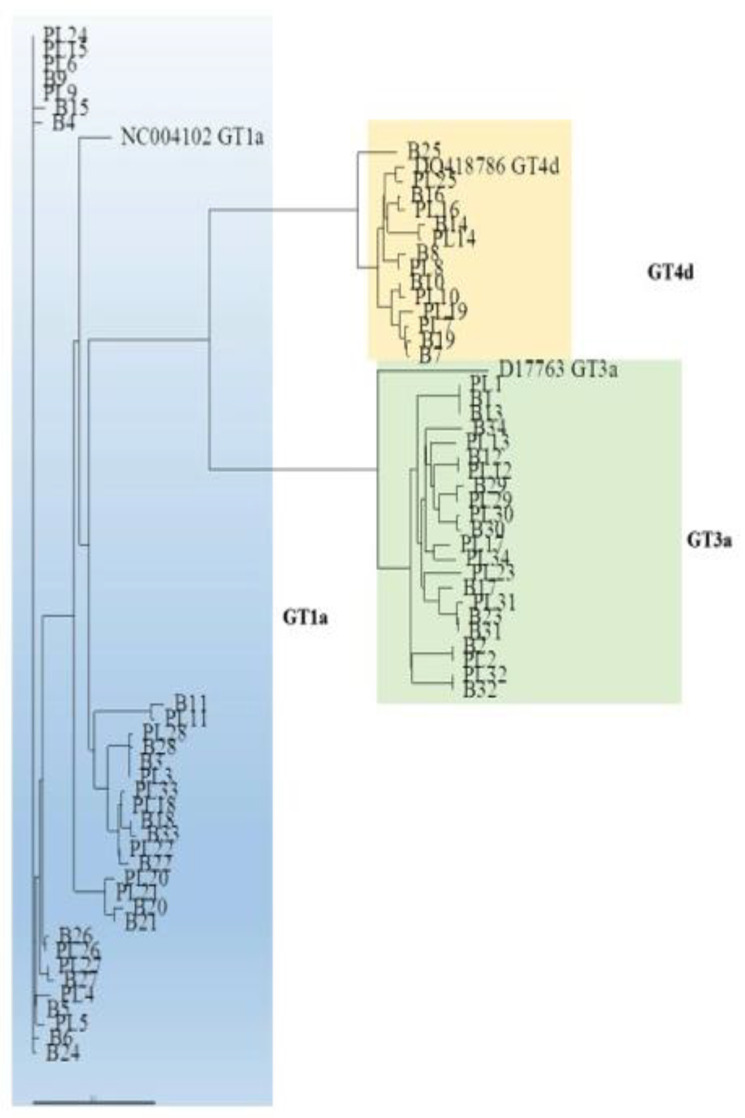
Phylogenetic tree of the NS3 domain in liver and plasma according to different HCV genotypes. Genetic distances were estimated by Kimura 2 parameters, and the phylogenetic tree was constructed by the neighbor-joining method using NS3 region sequences identified in the different compartments of 34 HIV/HCV coinfected patients: plasma (PL) and liver biopsy (B). The numbers refer to the nucleotide sequences derived from each patient. Sequences clustering within GT1a are delimited by a light blue rectangle, within GT3a by a green rectangle, and within GT4d by a yellow rectangle. The reference sequence for each genotype was retrieved from GenBank and added for phylogenetic analysis (NC004102 for GT1a; D17763 for GT3a; DQ418786 for GT4d).

This phenomenon was observed across HCV infecting genotypes (Figure 2). The NS3 sequences from the liver and plasma compartments clustered within the respective prototype, confirming the genotype identified in plasma samples by routine laboratory testing (Figure 2).

**Figure 3:**
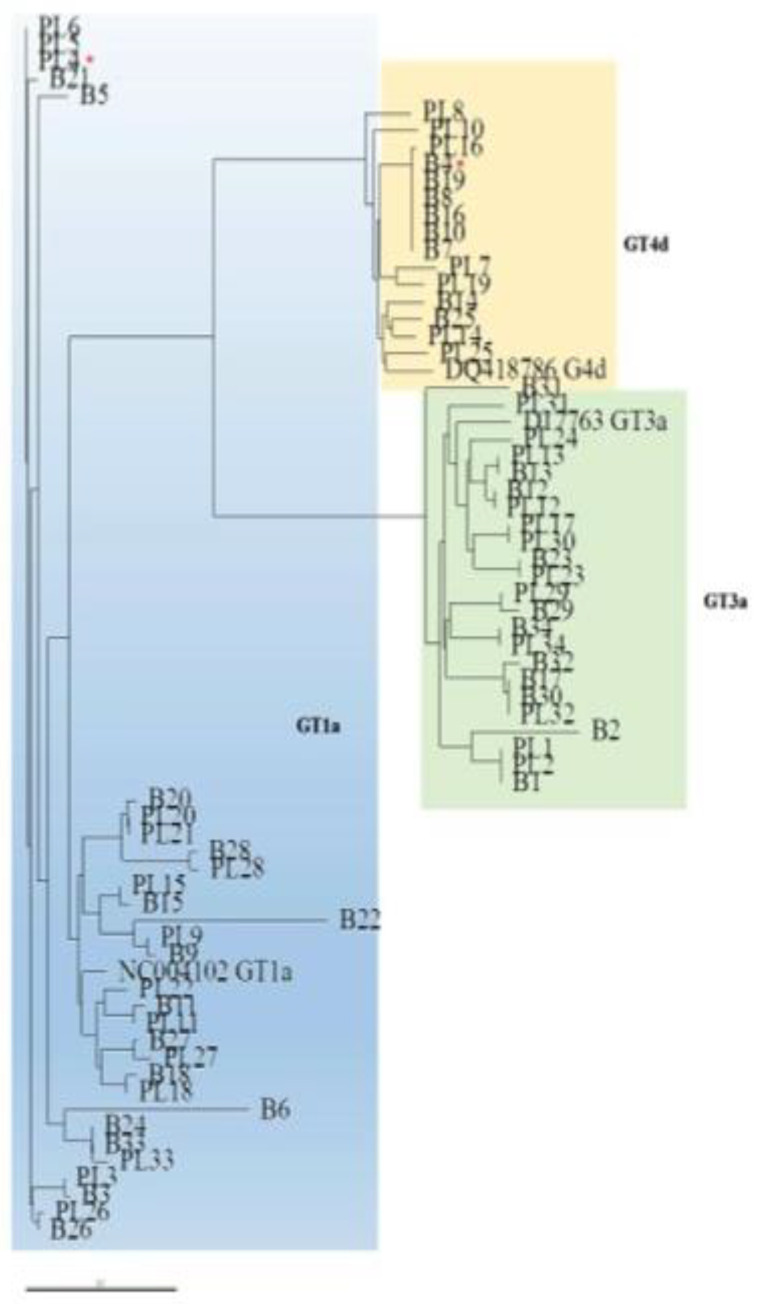
Phylogenetic tree of the NS5A domain in liver and plasma according to different HCV genotypes. Genetic distances were estimated by Kimura 2 parameters, and the phylogenetic tree was constructed by the neighbor-joining method using NS5A region sequences identified in the different compartments of 34 HIV/HCV coinfected patients: plasma (PL) and liver biopsy (B). The numbers refer to the nucleotide sequences derived from each patient. Sequences clustering within GT1a are delimited by the light blue rectangle, within GT3a by a green rectangle, and within Gt4d by a yellow rectangle. * The B4 sequence belonged to GT4d, but the corresponding PL4 sequence clustered within GT1a. The reference sequence for each genotype was retrieved from GenBank and added for phylogenetic analysis (NC004102 for GT1a; D17763 for GT3a; DQ418786 for GT4d).

Similarly, the phylogenetic analysis of the NS5A domain showed that paired plasma and liver sequences were not invariably closely related (Figure 3). The NS5A sequences from the liver and plasma compartments clustered within the respective prototype, with the exception of one case (PT4, see Figure 2 and Figure 3). In this patient, who was identified by NS3 and NS5A phylogenetic analysis of viral sequences from plasma samples as being infected with GT1a, the NS5A sequence obtained from the liver tissue clustered within GT4d and exhibited RAS T58P, a natural polymorphism in GT4d that confers resistance to the NS5A inhibitor ledipasvir.

#### 3.2.2. RAS Profiles According to HCV Infecting Genotype

A total of 11/34 (32.4%) patients had at least one RAS in the NS3 domain in the liver and/or plasma compartment. Concerning the presence of RASs in patients infected by GT1a, 8 of 16 (50%) patients had RAS Q80K in coupled plasma and liver samples. A difference in strain between liver and plasma was detected in three cases: PT6 had Q80K in the liver and a wild-type strain in the plasma, PT15 had Q80K in the plasma and the double mutant Q80K/D168H in the liver, and PT20 had Q80K in the plasma and a wild-type strain in the liver.

With regard to GT3a, a concordant strain with no RASs was detected in the plasma and liver of 11 (100%) patients.

The analysis of NS3 RAS profiles in the patients with GT4d showed that 7/7 (100%) patients carried a wild-type strain in the liver compartment, whereas a discordant strain from that detected in the liver was revealed in the plasma sample (D168S) of PT10 (Table 2). The comparison of the NS3 mutational profile (presence vs. absence of RASs) among different genotypes in the liver compartment showed that RASs were more frequently detected in GT1a (56.3% with respect to GT3a (0%) and GT4d (0%); *p* = 0.0010). Similarly, in the plasma compartment, GT1a exhibited a higher frequency of RASs (56.3%) compared to GT3a (0%) and GT4d (14.3%, *p* = 0.0043; Table 2).

Overall, 10/34 (29.4%) sequences had at least one RAS in the NS5A domain in the liver and/or plasma compartment. With regard to GT1a, 2/16 (12.5%) patients had NS5A RASs exclusively in the liver tissue: PT3 had triple mutant M28R/Q30R/L31R, and PT6 had double mutant M28L/Q30R (Table 2). Considering GT3a, all NS5A sequences were conserved other than that detected in PT2, showing amino acid substitution M28V in the liver but not in the corresponding plasma compartment. Concerning GT4d, NS5A RASs were revealed in 7/7 (100%) liver tissues and 5/7 (71.4%) plasma samples, and the RAS was invariably T58P (Table 2).

Comparison of the NS5A domain (presence or absence of RASs) between different genotypes in the two compartments showed that GT4d had a higher frequency of RASs (100%) compared to GT1a (12.5%) and GT3a (9.1%) in liver tissue (*p* = 0.0007). Similarly, GT4d had a higher frequency of RASs (71.4%) in the plasma compartment compared to GT1a (0%) and GT3a (0%, *p* < 0.0001).

With regard to the presence of RASs along the NS3 and NS5A domains, 19/34 (55.9%) patients had at least one RAS in NS3 and/or NS5A in the liver tissue and/or the plasma sample, with higher segregation of RASs in the liver (7 vs. 2 in the plasma).

#### 3.2.3. Subsequent Treatment with DAAs

Among 19 patients with RASs in the NS3 or NS5A domain in the liver or plasma compartment, 13 were subsequently treated with DAAs in the years 2015–2018, and all but one had an SVR. Unfortunately, other than for the patient with no response, a subsequent plasma sample was not available for virological investigation.

The patient (PT10) infected with GT4d who had plasma available at the time (May 2015) of treatment with DAAs (sofosbuvir plus ledipasvir) had the same RAS profile as detected in 2006 at both the baseline evaluation and treatment failure. At retreatment (April 2018) with voxylaprevir/sofosbuvir/velpatasvir, this patient maintained the same RAS profile but had an SVR.

## 4. Discussion

In the present study, sequence analysis of the NS3 protease domain and NS5A domain revealed a high frequency of natural RASs in HIV/HCV coinfected patients; 55.9% of patients had at least one RAS in the NS3 and/or NS5A domain in the liver tissue and/or plasma sample. The RASs were segregated into the liver rather than the plasma (7 vs. 2). Interestingly, the phylogenetic analysis of these regions showed that sequences from paired liver and plasma samples from each patient were not invariably closely related, and this phenomenon was observed for both domains across different genotypes. We do have a clear explanation for this finding. It is possible that the evolution of HCV isolates in different compartments is influenced by specific environmental conditions, rendering the HCV variants from the same compartment more closely related among patients than within patients. We detected a concordant dominant strain harboring the RAS Q80K, which confers resistance to protease inhibitor simeprevir, in half of the coupled liver tissue and plasma samples from patients infected with GT1a. Three (18.8%) patients (PT6, PT15, and PT20) had discordant NS3 sequences in the liver and plasma compartments: PT6 and PT20 had the mutant Q80K in the liver and plasma, respectively, whereas PT15 had double mutant Q80K/D168H in the liver, which confers resistance to simeprevir, cross-resistance to paritaprevir, and reduced susceptibility to grazoprevir. The NS3 domain in GT3a and GT4d was conserved in the liver and plasma compartments, other than one patient (PT10) infected with GT4d who exhibited segregation of a variant with aa substitution (D168S) in the plasma but not the liver (Table 2).

The comparison of NS3 RASs in different genotypes showed that GT1a was less conserved in the liver and plasma compartments with respect to GT3a and GT4d. This result suggests that, unlike GT1a, the NS3 region in GT3a and GT4d is not under selective pressure in the two compartments explored.

A previous study [14] using next-generation sequencing to investigate the compartmentalization of RASs within the NS3 of HIV-negative patients infected with HCV GT1b showed that an NS3 resistance-associated variant was dominant in 1/10 liver tissues and the corresponding plasma sample (10% frequency). However, they found minor variants harboring RASs at the intrahepatic level rather than in the plasma compartment. Via population sequencing, we showed a higher frequency of RASs around different genotypes (10/34, 29.4%) in the NS3 domain. Although population sequencing is less sensitive than next-generation sequencing for detecting minor variants, we found compartmentalization of the HCV strains harboring RASs in 4/34 (11.8%) patients.

In the present study, we included HIV/HCV coinfected patients, who have a higher prevalence and risk profile for HCV superinfection or mixed infection and/or HCV recurrence after eradication, rendering the virus more prone to developing within-host genetic diversity. Therefore, the different characteristics of patients and the HCV genotype are likely responsible for the discrepancy in the frequency and compartmentalization of NS3 RASs between the previous study (14, GT1b) and ours (GT1a, GT3a, GT4d).

With regard to the NS5A isolates, other than PT4 showing a different genotype in the plasma and liver in the NS5A domain, 10/34 (29.4%) sequences had RASs in the NS5A domain in the liver or plasma, and the compartmentalization of RASs was observed in five (14.7%) cases: two patients infected with GT1a, one with GT3a, and two with GT4d (Table 2). The two patients infected with GT1a had RASs in the liver but not the corresponding plasma sample. In the first case (PT3), the aa substitutions M28R and Q30P were detected, as was the RAS L31R that is associated in GT1a with specific resistance to elbasvir and ledipasvir. In the second case (PT6), the mutation M28L and RAS Q30R, which confer resistance to first-generation NS5A inhibitors, were detected. In GT3a-infected patients, all sequences were conserved in paired liver and plasma samples, other than in one case (PT2) with a mutation at a scored position (M28V) in the liver.

Interestingly, for GT4d, a dominant strain harboring T58P, which is associated with resistance to ledipasvir, was revealed in 7/7 (100%) liver tissues and 5/7 (71%) plasma samples, suggesting that this is a polymorphic site in subtype 4d.

A very recent study [16] of HIV-negative patients with severe liver disease (cirrhosis and/or hepatocellular carcinoma) showed 22% RAS compartmentalization using population (Sanger) sequencing of the NS3, NS5A, and NS5B domains across GT1a, GT1b, GT3a, and GT4d. Although we examined a different group of patients (all HIV/HCV coinfected, with less severe liver disease and paired specimens collected during years 2000–2006 when DAAs were not in use), we identified a similar frequency (26.5%) of RAS compartmentalization including the NS3 and NS5A domains in the analysis using the same method (Sanger). This finding suggests that the presence and compartmentalization of RASs is not a rare event in HIV/HCV coinfected patients harboring GT1a or GT4d, and it seems less frequent in those infected with GT3a.

Notably, we found discordance in genotype in the plasma and liver tissue of one patient (PT4) in the NS5A phylogenetic analysis, but not the NS3 phylogenetic analysis of the liver tissue, suggesting that this patient harbored an intergenotypic recombinant strain that was dominant in the liver but not the plasma compartment.

We did not perform a full sequence analysis of the HCV genome, though it is the more appropriate method for identifying recombinant forms [25]; therefore, we cannot exclude that this patient had a mixed infection with the dominance of a different genotype in the plasma and liver tissue.

The only patient (PT10) with sequential plasma available for virological investigation exhibited the naturally occurring RAS T58P at the time of his first treatment with DAAs (sofosbuvir/ledipasvir; anti-NS5b associated with anti-NS5A) in 2015 and at retreatment due to virological failure about 3 years later with voxylaprevir/sofosbuvir/velpatasvir, indicating good fitness for this variant resistant to anti-NS5A ledipasvir but not velpatasvir.

## 5. Limitations of the Study

Our study has certain limitations. As this was a retrospective study, we included only patients with paired liver and plasma samples available in the years 2000–2006. A number of patients were subsequently treated with DAAs, but plasma samples were not available for virological investigation before DAA therapy. Therefore, it was not possible to investigate the dynamic change in RASs and their possible impact on the treatment response to new antivirals.

Due to the retrospective design of the study, the analysis at the intrahepatic level was performed using paraffin-embedded liver tissue. Fresh-frozen tissue allows for the extraction of higher-quality nucleic acids than paraffin sections. However, most current PCR and sequencing applications typically require a small amount of nucleic acids, and the amplification of nucleotides is more easily achieved. In addition, we assessed the RNA quality by performing GAPDH amplification, and only PCR and sequencing assays confirmed in at least two different experiments were considered.

We applied population sequencing to reveal variants with RASs, which restricted the detection rate with respect to next-generation sequencing to those present at a rate of >15% in a patient. However, in clinical trials, only RASs present in >15% of the viral population have been shown to be clinically relevant. Thus, in clinical practice, HCV genotypic resistance testing can be carried out by population sequencing. As data regarding RAS compartmentalization testing are scarce but useful in an HIV/HCV coinfected group, this easy and less expansive biological approach (population sequencing) could contribute to the idea of testing liver biopsies in selected patients (e.g., patients with repeated treatment failure and advanced liver disease).

## 6. Conclusions

We found that the RAS profile within the NS3 protease domain could be associated with cross-resistance to different compounds of the same class at the intrahepatic level for GT1a, whereas GT3a and GT4d seemed to be more conserved with low or no compartmentalization of RASs.

With regard to the NS5A domain, the segregation of RASs was evident at the intrahepatic level not only in GT1a but also in GT4d. Taken together, our data suggest that HCV diversification within the liver may explain unsuccessful HCV treatment in certain patients, particularly those infected with GT1a, even in the era of new DAAs.

## Figures and Tables

**Table 1:** Characteristics of HIV/HCV coinfected patients in whom the NS3 and NS5A RASs were assessed.

Characteristic	Overall (*n* = 34)	GT1a (*n* = 16)	GT3a (*n* = 11)	GT4d (*n* = 7)	*p*
Age, years	40 (37–44)	40 (37–42)	39 (38–44)	42 (39–41)	0.553
Sex, male/female	24/10	13/3	7/4	4/3	0.419
Risk factor for HIV infection, IVDU/SC/unknown	21/8/5	9/3/4	8/3	4/2/1	0.495
Duration of HIV infection, years	13 (8–18)	13 (8–18)	12 (8–18)	16 (10–15)	0.839
ALT, U/L	72 (52–159)	66 (50–126)	129 (66–156)	64 (36–202)	0.598
AST, U/L	65 (45–126)	62 (42–108)	113 (53–126)	52 (46–139)	0.767
CD4 + T cell count, /mmc	479 (403–649)	479 (313–526)	466 (409–678)	562 (419–795)	0.509
CD8 + T cell count, /mmc	1047 (770–1191)	1048 (785–1227)	1058 (898–1114)	813 (720–1174)	0.922
HBsAg, pos/neg	2/32	1/15	1/10	0/7	0.724
HCV-RNA, log IU/mL	5.85 (5.33–6.16)	5.51 (4.18–5.88)	5.85 (5.51–6.15)	6.0 (5.7–6.28)	0.188
HIV-RNA, log copies/mL	2.63 (1.69–3.87)	1.91 (1.69–3.70)	2.77 (1.79–4.08)	3.7 (2.3–4.1)	0.484
Fibrosis degree (F0–F2/F3–F4) according to Metavir score	27/7	12/4	10/1	5/2	0.508
Cirrhosis, yes/no	2/32	1/15	0/11	1/6	0.724

RNA limit of detection, 12 IU/mL (1.07 log IU/mL). HIV-RNA limit of detection, 50 copies/mL (1.69 log copies/mL). NS5A RAS profiles were investigated in the liver and plasma according to HCV infecting genotype. Data are given as the number of patients or median (interquartile range). Abbreviations: IVDU = intravenous drug user; SC = sexual contact; HBsAg = hepatitis B surface antigen. Normal value for alanine aminotransferase (ALT) levels, <60 U/L; normal value for aspartate aminotransferase (AST) levels, <36 U/L.

**Table 2:** Characteristics of 19 HIV/HCV-infected patients with NS3 or NS5A RASs in the plasma and/or liver.

Patient	Sex	Risk Factor for HIV-1 Infection	Fibrosis Degree	HCV GT	HCV-RNA Log (IU/mL)	RAS NS3 Liver	RAS NS3 Plasma	RAS NS5A Liver	RAS NS5A Plasma
PT 3	F	IVDU	F0	1a	6.18	S	S	M28R-Q30P-L31R	S
PT 4 #	M	Unknown	F1	1a/4d	5.82	Q80K	Q80K	T58/*p*	S
PT 5	M	IVDU	F2	1a	3.96	Q80K	Q80K	S	S
PT 6	M	SC	F2	1a	5.89	Q80K	S	M28L-Q30R	S
PT 9	M	Unknown	F2	1a		Q80K	Q80K	S	S
PT 15	M	IVDU	F0	1a	5.85	Q80K-D168H	Q80K	S	S
PT 20	F	SC	F0	1a	5.46	S	Q80K	S	S
PT 21	M	IVDU	F1	1a	4.93	Q80K	Q80K	S	S
PT 24	M	IVDU	F3	1a	4.18	Q80K	Q80K	S	S
PT 26	M	ND	F3	1a	3.15	Q80K	Q80K	S	S
PT 27	F	IVDU	F1	1a	4.18	Q80K	Q80K	S	S
PT 2	M	SC	F1	3a	5.85	S	S	M28V	S
PT 7	F	SC	F1	4d	5.54	S	S	T58P	S
PT 8	F	SC	F1	4d	6.12	S	S	T58P	T58P
PT 10	M	IVDU	F2	4d	6.44	S	D168S	T58P	T58P
PT 14	M	IVDU	F3	4d		S	S	T58P	T58P
PT 16	M	IVDU	F0	4d	5.97	S	S	T58P	T58P
PT 19	F	Unknown	F2	4d	5.53	S	S	T58P	S
PT 25	M	IVDU	F4	4d	6.51	S	S	T58P	T58P

Abbreviations: PT = patient; F = female; M = male; IVDU = intravenous drug user; SC = sexual contact; GT = genotype. # PT4 harbored GT4d in the liver compartment in the sequence analysis of NS5A but GT1a in the NS3 analysis. HCV-RNA limit of detection, 12 IU/mL (1.07 log IU/mL).

## Data Availability

Not Applicable.
